# Malaria Morbidity in High and Seasonal Malaria Transmission Area of Burkina Faso

**DOI:** 10.1371/journal.pone.0050036

**Published:** 2013-01-08

**Authors:** Alphonse Ouédraogo, Alfred B. Tiono, Amidou Diarra, Souleymane Sanon, Jean Baptiste Yaro, Esperance Ouedraogo, Edith C. Bougouma, Issiaka Soulama, Adama Gansané, Amathe Ouedraogo, Amadou T. Konate, Issa Nebie, Nora L. Watson, Megan Sanza, Tina J. T. Dube, Sodiomon Bienvenu Sirima

**Affiliations:** 1 Centre National de Recherche et de Formation sur le Paludisme, Ouagadougou, Burkina Faso; 2 The EMMES Corporation, Rockville, Maryland, United States of America; 3 Groupe d'action et de Recherche en Santé, Ouagadougou, Burkina Faso; World Health Organization, Switzerland

## Abstract

**Background:**

Malariometric parameters are often primary endpoints of efficacy trials of malaria vaccine candidates. This study aims to describe the epidemiology of malaria prior to the conduct of a series of drug and vaccine trials in a rural area of Burkina Faso.

**Methods:**

Malaria incidence was prospectively evaluated over one year follow-up among two cohorts of children aged 0–5 years living in the Saponé health district. The parents of 1089 children comprising a passive case detection cohort were encouraged to seek care from the local health clinic at any time their child felt sick. Among this cohort, 555 children were randomly selected for inclusion in an active surveillance sub-cohort evaluated for clinical malaria during twice weekly home visits. Malaria prevalence was evaluated by cross-sectional survey during the low and high transmission seasons.

**Results:**

Number of episodes per child ranged from 0 to 6 per year. Cumulative incidence was 67.4% in the passive and 86.2% in the active cohort and was highest among children 0–1 years. Clinical malaria prevalence was 9.8% in the low and 13.0% in the high season (p>0.05). Median days to first malaria episode ranged from 187 (95% CI 180–193) among children 0–1 years to 228 (95% CI 212, 242) among children 4–5 years. The alternative parasite thresholds for the malaria case definition that achieved optimal sensitivity and specificity (70–80%) were 3150 parasites/µl in the high and 1350 parasites/µl in the low season.

**Conclusion:**

Clinical malaria burden was highest among the youngest age group children, who may represent the most appropriate target population for malaria vaccine candidate development. The pyrogenic threshold of parasitaemia varied markedly by season, suggesting a value for alternative parasitaemia levels in the malaria case defintion. Regional epidemiology of malaria described, Sapone area field centers are positioned for future conduct of malaria vaccine trials.

## Introduction

Despite suggestion of a general decline in malaria burden in sub-Saharan Africa, Burkina Faso has shown no evidence of decrease in high year-round endemicity; health facility reports in fact suggest an increase nationally from an estimated 1 million in the year 2006 to 4 million in 2009 [1]–[4]. Malaria prevention in the region is challenged by an underdeveloped health care system and inadequate coverage of effective drugs and vector control tools. Development of an effective malaria vaccine as an additional tool in the arsenal against malaria is widely recognized an important strategy for reduction in global malaria burden. Several malaria vaccines candidates in various stages of development have shown favourable safety and immunogenicity profiles [5].

Development and evaluation of malaria vaccine trials demands knowledge of the regional epidemiology of malaria; in fact malaria infection, clinical malaria, and mortality rates have represented primary endpoints of phase 2b or 3 of malaria vaccine trials [6]. Active and passive surveillance methods have more recently been proposed for the evaluation of malaria vaccine efficacy. Consensus guidelines [7] recognize the value of active case detection (ACD) in minimizing the influence of health care-seeking behaviour and allowing inference of age-associated and temporal patterns of disease. Limited to those seeking medical care, passive case detection (PCD) methods are likely to underestimate population burden of malaria but may provide information of greater relevance to a health services perspective [8].

Current malaria epidemiology data in endemic regions are needed to evaluate the public health significance of alternative interventions and to monitor effects of malaria control activities over time. We sought to describe the epidemiology of malaria prior to the conduct of a series of drug and vaccine trials by combing active and passive case detection of malaria episodes among children under five years living in a rural area of Burkina Faso.

## Patients, Materials and Methods

### Study area and population

Enrollment was initiated in January 2007 in the Saponé health district in the Bazèga province where the Centre National de Recherche et de Formation sur le Paludisme (CNRFP) has maintained a continuous demographic surveillance system (DSS) since 2005. The Sapone DSS is located in the centre of Burkina Faso, 40 km at the south from the capital, Ouagadougou in the Sudan-Sahelian eco-climatic zone (isohyets 600–800). The DSS covers an area of 1700 Km2 within a plateau dissected by the Nazinon river (White Volta river). Mean temperature recorded during 2007 was 29°C and total rainfall for the same period was 713 mm. The climate is characterized by a rainy season from June to September and dry season from October to May. The total resident population is estimated as 100,000 inhabitants and infant and children mortality is 105.3 per 1000 live births. Children below five years represent 19% of the population and total fertility rate is an estimated 45‰. The predominating ethnic groups in the area are Mossi and Fulani. Most of the population depends on subsistence farming of millet and domestic animals (poultry, cattle etc.). Houses are typically made of mud walls and grass or corrugated iron roofs [9]. One reference district hospital (CMA de Saponé with 32 beds) and 14 peripheral health facilities constitute the government health network in the district.

Study randomization was performed at two levels to achieve target sample sizes of PCD and ACD cohorts: i) two of the 14 peripheral health centres catchment areas composed of 13 villages were randomly selected, ii) 9 villages of the 13 villages were randomized to conduct passive case detection and 4 to conduct active case detection of malaria over one year follow-up. All 13 villages were situated within ≈7.5 km of the peripheral health centres, facilitating access to each PCD surveillance system.

Malaria transmission in the region is markedly seasonal and intense during the rainy season. The entomological inoculation rate is estimated at 0.3 and 44.4 infective bites/person/month during the dry and rainy seasons, respectively [10]. The main malaria vectors are *Anopheles gambiae*, *An. Arabiensis* and *An funestus. P falciparum* accounts for a 90% of malaria infections. Oral chloroquine was still in use in the communities during the study period despite parasite resistance of 57% (Sirima and al. unpublished data). The first line treatment for uncomplicated malaria in Burkina is now Coartem® or Artesunate Amodiaquine. In the study region these medications are distributed primarily by peripheral health centres; there is no private clinic or pharmacy alternative although prohibited or sub-standard antimalarials are sold from the local market. In 2007, bed net coverage was less than 5% of the DSS population, reflecting the absence of large scale bed net distribution in the region.

### Study design and subjects

Malaria surveillance over one year follow-up consisted of passive case detection among all patients attending the outpatient clinic; and active case detection by twice weekly home visits and two cross-sectional surveys among selected villages. The first cross sectional survey occurred during low malaria transmission season and the second during high transmission season. Overall and age-specific incidence of clinical malaria were compared among the passive and active cohorts.

All children aged under five years and living for at least three months in the study area were eligible for inclusion in either cohort at study initiation. Exclusion criteria were presence of major congenital defect, chronic disease or severe anaemia (haemoglobin value less than 6 g/dl); these conditions were considered to increase participant risks associated with blood draws, and potentially require absence from the study area for receipt of specialized care. An age-stratified sample was generated from the updated census of Saponé demographic surveillance system area. Five age-specific age strata (0–11, 12–23, 24–35, 36–47 and 48–59 months) included 245, 201, 225, 219 and 199 children respectively in the PCD, and 131, 103, 113, 115 and 93 children in the ACD cohort. Children under one year were recruited quarterly to maintain balance among cohorts. Signed or thumb-printed (in the presence of an impartial witness) written informed consent from child's parent/legal guardian was required prior to inclusion in the study. Each child was provided with an identity card and the caregiver was given instructions how to access health centre in the event of illness.

#### Passive surveillance

In January 2007 the PCD cohort enrolled a total of 1090 children from 9 villages in the catchment areas of two health facilities. In the two community clinics providing routine care and in the Saponé District Hospital emergency unit, local health staff received training in study procedures and were provided with study case report forms to record clinical information of children enrolled in either cohort. At each attendance to a health facility (community clinics or Saponé District Hospital), study children received a brief physical examination and parents reported previous health events. In the event of reported history of fever within 24 hours or measured axillary temperature ≥37.5°C, a finger-prick blood sample was collected for preparation of duplicate thick blood smears. One slide was read immediately (within two hours) in the Saponé District Hospital and each transferred to the CNRFP lab for quality assurance. Rapid diagnosis tests (RDT) were performed in the two community clinics of the study area where an operating lab was not present. Results were used to guide diagnosis and prompt adequate management of malaria cases. Antimalarial medication was provided as needed at no cost to study participants. This system operated 24 hours per day each day of the year to continuously ascertain malaria episodes among presenting study children. Free drug treatment was made available as indicated for malaria and non-malaria illnesses.

#### Active surveillance

The active surveillance cohort consisted of 555 children from four randomly selected villages who were visited in the home twice per week for detection of clinical malaria episodes over one year follow up. The target sample size was achieved after recruitment of children living in the first four of thirteen villages randomly selected for potential study inclusion.

Trained fieldworkers visited the children in their homes twice a week on the same week day and before 11:00 AM in order to minimize the bias due to diurnal variation of the body temperature [11]. A morbidity questionnaire was administered for evaluation of fever history and medication use. Respondents to the questionnaire were the children's parents/guardians who were aged ≥18 years and were at home at the time of the visit. A brief physical examination was performed and axillary temperature recorded. In the event of repeated fever in the past 24 hours or axillary temperature ≥37.5°C, a finger prick blood sample was collected for preparation of thick blood smears for microscopic examination. Feverish children received a malaria rapid diagnostic test for prompt diagnosis and treatment. All symptomatic children were referred to the nearest local heath staff or to the Saponé District Hospital to receive adequate treatment and follow-up as clinically appropriate at no cost to the participant. Between the two scheduled field-worker visits, parents were encouraged to report to the nearest community clinic or hospital at any time whenever their children showed signs of sickness. Children were provided with study identification cards for presentation during clinical or hospital attendance.

Diagnostic procedures were repeated during two seasonal cross sectional surveys, which contributed to analyses of prevalence of malariometric indicators and malaria attributable fraction of fevers. The first cross sectional survey occurred during the dry season and the second one during the wet season of the same year.

### Laboratory methods

Thick and thin blood films were prepared, air-dried, fixed, Giemsa-stained, and examined using a light microscope fitted with 100× oil immersion lens. Number of parasites and leucocytes were counted to reach 200 leucocytes for positive slides. Slides were declared negative only after 100 high power fields has been read. Parasite numbers were converted to a count/µL. All slides were read twice and slides with discrepant result were then read a third time. Haemoglobin was measured at each survey using a portable Hemocue.

### Study Definitions

Fever was defined as axillary temperature ≥37.5°C and or reported fever within the previous 24 hours. A clinical malaria episode was defined as an axillary temperature of ≥37.5°C or history of fever in last 24 hours, and positive asexual parasitemia (*P. falciparum* trophozoite count greater than 0). Analyses of clinical malaria incidence were repeated using the alternative parasitemia thresholds of >0, ≥2500, and ≥5000.

In analyses of clinical malaria incidence, children were censored for 28 days after recording an episode, to ensure that the infection causing the episode was only recorded once.

### Statistical analysis

Overall and age-specific fever and malaria incidence rates over one year follow-up were calculated as the number of events malaria cases divided by the total person time at risk. Cumulative incidences were estimated by the Kaplan-Meier method. Children were censored for each day he or she recorded a fever or for 28 days after recording an episode, to ensure that the infection causing the episode was only recorded once. In analyses of both fever and clinical malaria incidence, children were censored after 365 days of follow-up, or at the date of death/out migration (N = 25). Those who did not experience death/migration but were followed for less than one year were censored at the last recorded passive case detection visit (Febuary 29^th^ 2008). The low transmission season was defined as the date of first enrollment (January 23^rd^ 2007) through May 31^st^ 2007 and November 1^st^ 2007 through date of last observation (February 29^th^ 2008). The high transmission season was defined as June 1^st^ 2007 through Oct 31^st^ 2007.

Malaria attributable fraction of fevers, and sensitivity and specificity of alternative parasite thresholds for the malaria case definition were calculated according to methods described by Dicko et al [12] Briefly, the odds ratio for the risk of fever associated with increasing parasite density was estimated separately for each transmission season, using data from the first and second cross-sectional surveys. The model was of the form: *Log(p_i_/(i−p_i_)) = β_o_+β_1_x_i_^τ^*, where *p_i_* is the fitted probability that individual *i* is a fever case rather than a control, and *x_i_^τ^* is the parasite density to the power of *τ*. Several models were fit using alternative *τ* values, and a final *τ* was selected as the value that provided the model of best fit. Attributable fraction, sensitivity and specificity were then defined as detailed by Smith et al [13].

Attributable fraction estimates have been found to vary depending on whether fever cases are defined using objective (temperature ≥37.5°C) or both objective and subjective fever [14]. To compare estimates obtained by each method, we repeated the analyses using the following alternative definitions of fever. Method 1: A fever case was defined as axillary temperature ≥37.5 or reported history of fever in past 24 hours; a control was defined as no axillary temperature ≥37.5 or reported history of fever in past 24 hours. This analysis included 522 and 487 children who had fever and parasitemia data at the first and second cross-sectional surveys, respectively. Method 2: A fever case was defined as axillary temperature ≥37.5; a control was defined as no axillary temperature ≥37.5 or reported history of fever in past 24 hours. Of the children who had fever and parasitemia data at each survey, this analysis excluded those who did not meet either the definition of a fever case or control, as they had no axillary temperature ≥37.5 but had reported history of fever in past 24 hours. This exclusion yielded a final sample size of 494 and 447 children at the first and cross-sectional surveys, respectively. While reduction in sample size may be seen as a disadvantage of relying exclusively on objective fever data, this approach offers the potentially important advantage of reducing misclassification of fevers due to errors in reporting.

Sample sizes expected to yield robust estimates of malaria incidence were calculated using Epi Info version 6 assuming weekly incidence densities estimated in previous years from regional DSS and targeted surveillance within one of the study site villages. Access was used for double data entry and query resolution and SAS 9.2 for data analysis.

The spatial analysis map of community clinic accessibility was produced using Arc View GIS 3.2.

### Quality assurance

Validity and completeness of data collection procedures were reviewed weekly by a team of physician researchers. In the ACD cohort, each week a sample of fifteen (average 50 children) randomly selected households were revisited by a field supervisor. The supervisor interviewed the parents/guardian to verify reporting of fever or collection of a blood sample.

All slides were read twice independently; if the ratio of densities from the first two readings was >1.5 or <0.67 or if <30 parasites were counted with a difference in the number of parasites >10, the slide was evaluated a third time. The definitive result was the geometric mean of the parasite density. In the event of discrepancy the slide was evaluated a third time and the final result defined as the majority reading for positivity.

Data queries were generated weekly to identify and resolve missing or erroneous values.

### Ethics Statement

The study protocol and the informed consent form were approved by the Burkina Faso ministry of health ethic committee. The study was conducted in compliance with principles set out by the International Conference on Harmonization Good Clinical Practices, the Declaration of Helsinki and the regulatory requirements of Burkina Faso. Individual written informed consent was obtained from all children's parents or legal representatives, in the presence of an independent witness for illiterate parents/legal representative. The conduct of the study was monitored by the sponsor.

## Results

### Population characteristics

Of 1644 children enrolled, analyses include 1089 (66.3%) in PCD and 555 (33.7%) in ACD. Eight (8) children died and 17 (1%) withdrew during the follow up period. Enrolment within each cohort was approximately equally distributed among gender and age groups (Table 1).

**Table 1:** Characteristics of children at recruitment.

	Cohort C	Cohort B
Gender M	289 (52.1%)	551 (50.6%)
F	266 (47.9%)	538 (49.4%)
Age group (years)		
[0–1]	131 (24%)	245 (23%)
[>1–2]	103 (18%)	201 (18%)
[>2–3]	113 (20%)	225 (21%)
[>3–4]	115 (21%)	219 (20%)
[>4–5]	93 (17%)	199 (18%)
N	555 (100%)	1089 (100%)

### Median number of visits by child

ACD children where present during 55279 of 57720 (95.8%) home visits attempted. Losses to follow up were due to parents moving away from the DSS area. Median number of visits per child was 100 (range 1–104). ACD children presented to the community clinic without reference 3389 times; median number of visits per child was 6 (range 1–20).

PCD children contributed 5742 visits; median number of visits per child was 5 (range 1–14).

### Incidence of fever

ACD scheduled & unscheduled visits identified 2668 cases of fever; 79% (n = 2129) of these were detected through unscheduled visits. 2382 cases of fever were detected by passive surveillance visits.

In both ACD and PCD cohorts fever was more frequent among infants and markedly lower among children aged 4–5 years (Table 2). Fever incidence also varied substantially by malaria transmission season: episodes per child-year-at-risk (95% CI) were 1.6 (1.5–1.7) (PCD) and 2.3 (1.9–2.7)(ACD) during the low season, versus 3.2 (3.0–3.4) (PCD) and 7.0 (6.7–7.4) (ACD) in the high season.

**Table 2:** Age-specific incidence of fever over one year follow-up.

Age group (years)	Number of children	Number of visits	Number of fevers	Total p-yrs* at risk	Incidence rate (95% CI) (per p-yr at risk)
ACTIVE SURVEILLANCE					
[0–1]	131	11117	789	112.4	7.0 (6.5, 7.5)
[>1–2]	103	10244	588	101.3	5.8 (5.3, 6.3)
[>2–3]	113	11733	558	111.4	5.0 (4.6, 5.4)
[>3–4]	115	12388	456	113.1	4.0 (3.7, 4.4)
[>4–5]	93	9797	277	92.2	3.0 (2.7, 3.4)
Total	555	55279	2668	530.5	5.0 (4.8, 5.2)
PASSIVE SURVEILLANCE					
[0–1]	245	1738	813	216.5	3.8 (3.5, 4.0)
[>1–2]	201	1287	569	198.2	2.9 (2.6, 3.1)
[>2–3]	225	1121	474	220.1	2.2 (2.0, 2.4)
[>3–4]	219	863	291	217.4	1.3 (1.2, 1.5)
[>4–5]	199	733	235	195.5	1.2 (1.1, 1.4)
Total	1089	5742	2382	1047.7	2.3 (2.2, 2.4)

### Incidence of clinical malaria episodes

Incidence of clinical malaria defined by alternative parasitemia thresholds was nearly two fold higher in the ACD relative to the PCD cohort over one year follow up. *P. falciparum* was identified among 1071 (40%) of 2668 fevers in the ACD, and among 1208 (51%) of 2382 fevers in the PCD cohort. Clinical malaria incidence was higher in younger age groups among both the ACD and PCD cohorts (Table 3). Number of episodes per child ranged from 0 to 6.

**Table 3:** Age-specific incidence of clinical malaria episodes parasitemia threshold for malaria case definition: >0 parasites/µl during high and low seasons.

Age group (years)	Number of children	Number of visits	Number of episodes	Total p-yrs* at risk	Incidence rate (95% CI) (per p-yr at risk)	Cumulative incidence
PASSIVE SURVEILLANCE						
[>0–1]	245	1738	340	192.4	1.8 (1.6, 2.0)	83.6
[>1–2]	201	1287	291	176.9	1.6 (1.5, 1.8)	69.1
[>2–3]	225	1121	267	200.8	1.3 (1.2, 1.5)	67.9
[>3–4]	219	863	169	205.2	0.8 (0.7, 1.0)	41.6
[>4–5]	199	733	141	185.1	0.8 (0.6, 0.9)	55.9
Total	1089	5742	1208	960.4	1.3 (1.2, 1.3)	67.4
ACTIVE SURVEILLANCE						
[0–1]	131	11117	243	95.5	2.5 (2.2, 2.9)	93.0
[>1–2]	103	10244	230	85.1	2.7 (2.4, 3.1)	89.6
[>2–3]	113	11733	260	92.7	2.8 (2.5, 3.2)	94.0
[>3–4]	115	12388	215	97.5	2.2 (1.9, 2.5)	80.7
[>4–5]	93	9797	123	83.3	1.5 (1.2, 1.8)	71.4
Total	555	55279	1071	454.1	2.4 (2.2, 2.5)	86.2

Incidence of clinical malaria defined by the alternative parasitemia threshold of 5000 parasites/µL (episodes per child-year-at-risk) (95% CI) was 1.6 (1.5–1.7) in the ACD and 0.8 (CI 0.8–0.9) in the PCD cohort (Table4).

**Table 4:** Incidence density of malaria episode according to season and different level of parsitemia.

Stratum	Number of observations	Number of children having at least one episode (>0 parasites/µl)	Incidence rate per year at risk >0 parasite/µL	Incidence rate per year at risk >2500 parasite/µL	Incidence rate per year at risk >5000 parasite/µL
**Active cohort**	55279	480	2.4 (2.2, 2.5)	1.8 (1.7, 1.9)	1.6 (1.5, 1.7)
**Passive cohort**	5742	702	1.3 (1.2, 1.3)	0.9 (0.9, 1.0)	0.8 (0.8, 0.9)
**Active cohort high season**	22013	424	3.9 (3.6, 4.2)	3.2 (2.9, 3.4)	2.9 (2.7, 3.2)
**Passive cohort high season**	1824	585	2.2 (2.1, 2.4)	1.8 (1.6, 1.9)	1.6 (1.5, 1.7)
**Active cohort low season**	33266	304	1.4 (1.3, 1.6)	0.9 (0.8, 1.0)	0.8 (0.7, 0.9)
**Passive cohort low season**	3918	323	0.7 (0.6, 0.7)	0.4 (0.4, 0.5)	0.3 (0.3, 0.4)

Clinical malaria incidence was higher in younger children within each season and among alternative parasite thresholds for malaria case definition. Children aged ≥4 years experienced fewest malaria episodes with incidence rates ranging between 0.8 and 2.2 episodes per child-year-at-risk. This pattern was apparent by each method of incidence assessment (Table 3).

**Figure 1:**
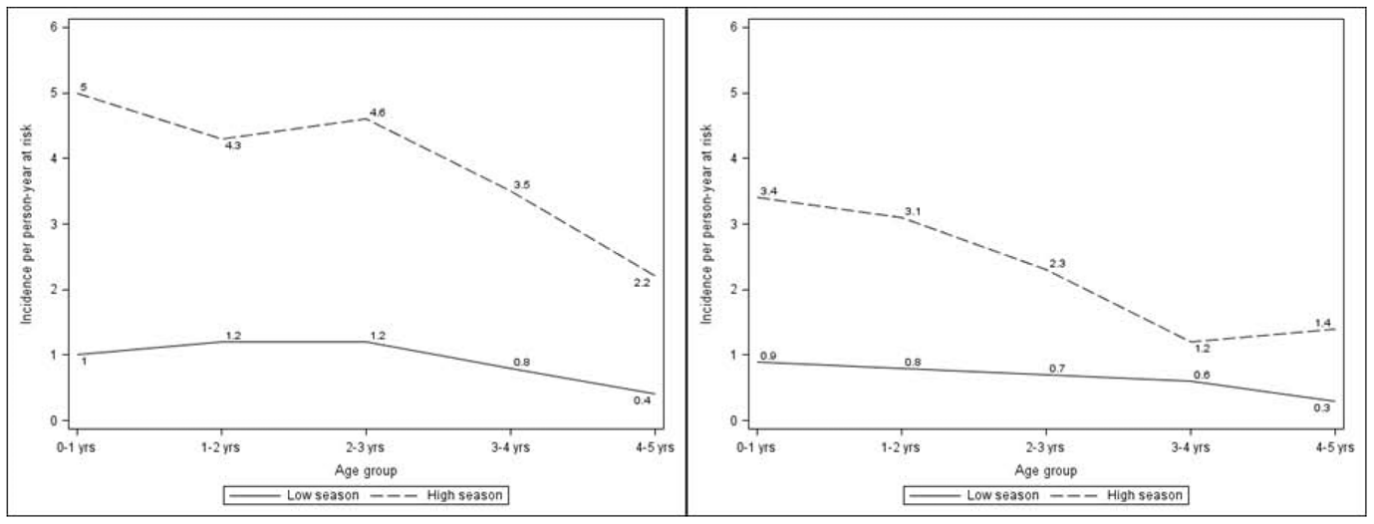
Age-specific malaria incidence according to the season. a. Seasonal variation in age-specific clinical malaria incidence evaluated by active surveillance over one year. b. Seasonal variation in age-specific clinical malaria incidence evaluated by passive surveillance over one year.

Among all age groups clinical malaria incidence was highest following the peak rainfall (Figure 1a & 1b).

### Malariometric parameters according to the season

Two cross-sectional surveys among children in the ACD cohort evaluated prevalence of malariometric indices within the low and high transmission seasons (Table 5).

**Table 5:** Malariometric parameters Prevalence by Age Group in active surveillance cohort according to the season.

Age group (years)	Clinical malaria	*P. falciparum* infection	Gametocyte carriage	Splenomegaly	Hemoglobin mean (SD)
Low Season					
[0–1]	6 (5.7%)	24 (22.9%)	15 (14.3%)	4 (3.8%)	9.58 (2.13)
[>1–2]	10 (9.7%)	53 (51.5%)	30 (29.1%)	15 (14.6%)	9.06 (1.64)
[>2–3]	12 (10.6%)	74 (66.7%)	40 (36.0%)	18 (15.9%)	9.51 (1.31)
[>3–4]	15 (13.2%)	78 (70.3%)	36 (32.4%)	32 (28.1%)	9.88 (1.53)
[>4–5]	9 (9.7%)	71 (77.2%)	33 (35.9%)	19 (20.7%)	10.58 (1.16)
Total (N = 528)	52 (9.8%)	300 (57.5%)	154 (29.5%)	88 (16.7%)	9.71 (1.66)
High Season					
[0–1]	15 (16.1%)	35 (37.6%)	6 (6.5%)	4 (4.3%)	9.59 (1.18)
[>1–2]	15 (16.5%)	49 (53.8%)	23 (25.3%)	6 (6.6%)	10.13 (1.30)
[>2–3]	17 (16.3%)	49 (47.1%)	12 (11.5%)	12 (11.7%)	10.73 (1.09)
[>3–4]	8 (7.1%)	61 (54.5%)	29 (25.9%)	14 (12.5%)	10.93 (1.19)
[>4–5]	8 (9.4%)	45 (52.9%)	12 (14.1%)	4 (4.7%)	11.11 (1.12)
Total (N = 485)	63 (13.0%)	239 (49.3%)	82 (16.9%)	40 (8.3%)	10.53 (1.29)

**Figure 2:**
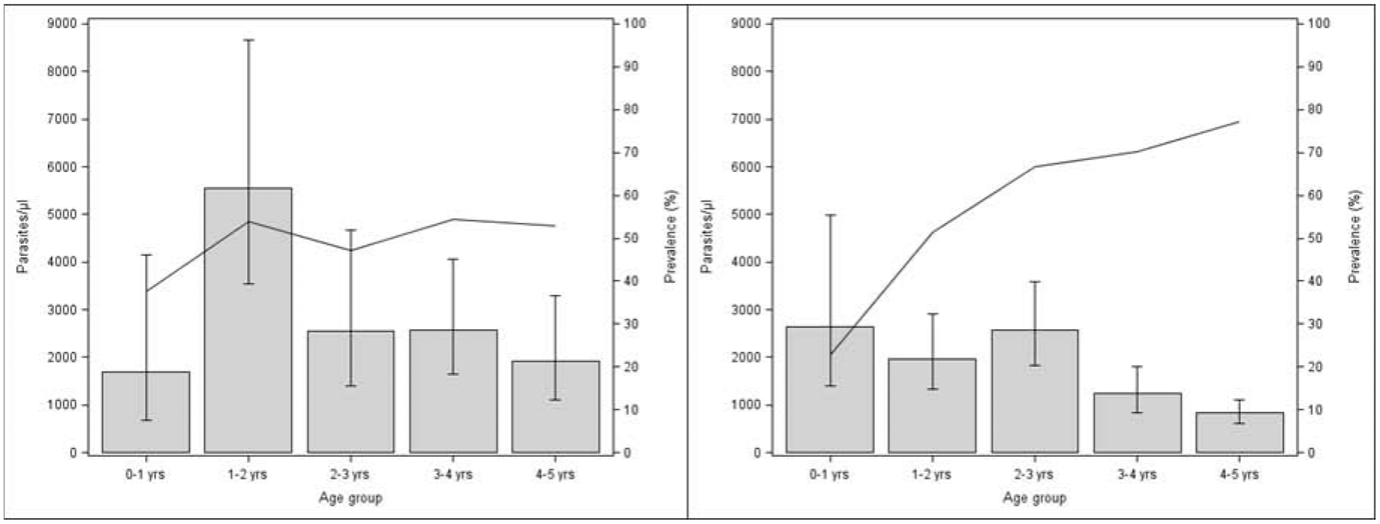
Prevalence of parasitemia and geometric mean parasite densities according to the season. a. Age-specific prevalence of parasitemia and geometric mean parasite densities in the high transmission season. Line indicates parasitemia prevalence. Bars indicate geometric parasite density and 95% confidence interval. b. Age-specific prevalence of parasitemia and geometric mean parasite densities in the low transmission season. Line indicates parasitemia prevalence. Bars indicate geometric parasite density and 95% confidence interval.

Clinical malaria prevalence was 9.8% in the low versus 13.0% in the high season (p = 0.12); prevalence of asexual *P. falciparum* infection was 57.5% in the low versus 49.3% in the high season (p = 0.01). Geometric mean parasite density (parasites/µL) (95% CI) was lower during the low relative to high season: 1554 (1307.5–1848.9) versus 2673.2 (2077.1–3440.4) (p<0.01) (Figure 2a & 2b).

### Time to first malaria episode at enrollment

**Figure 3:**
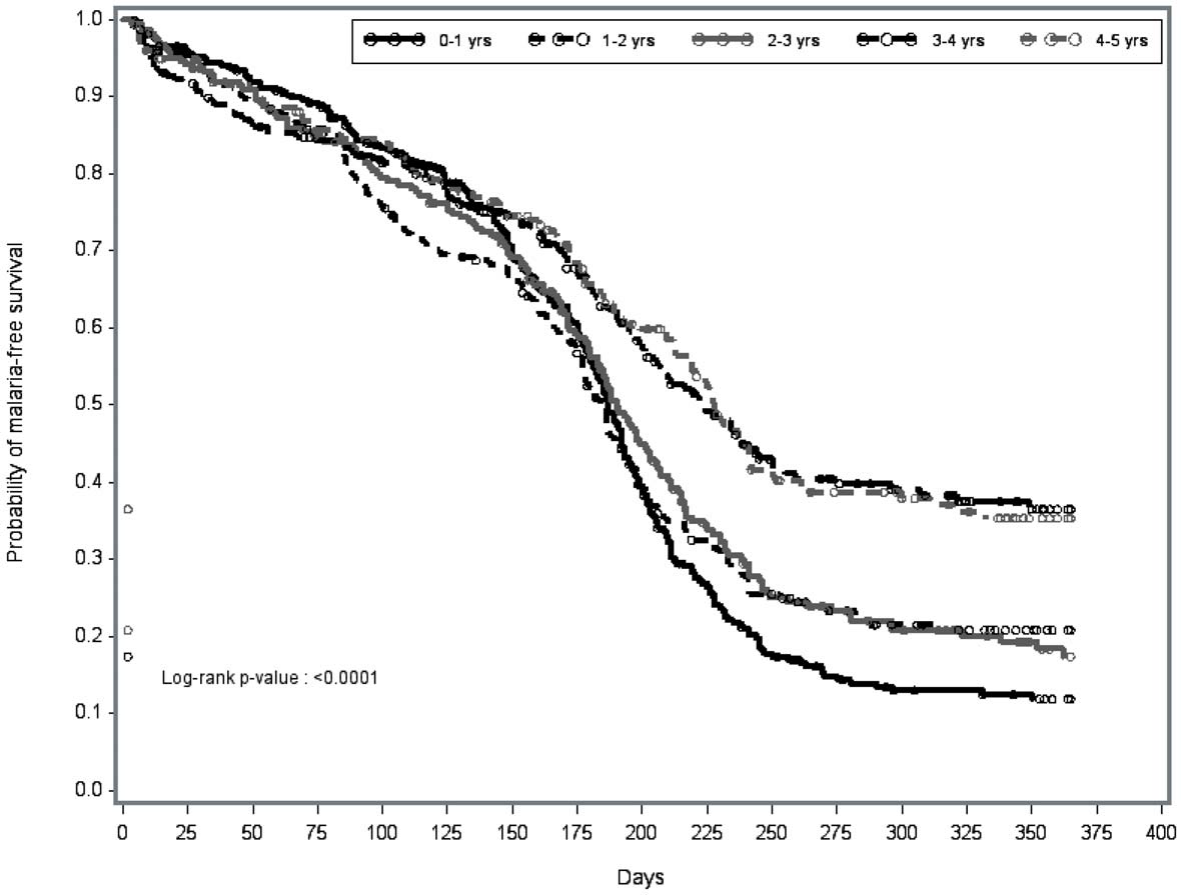
Time to first malaria episode among children 0–5 years evaluated by active surveillance from the low through high transmission season.

Median days to first malaria episode ranged from 187 (95% CI 180–193) among children 0–1 years to 228 (95% CI 212, 242) among children 4–5 years in the ACD cohort. Cumulative incidence among these age groups ranged from 93%–71% (Figure 3).

### Malaria attributable fraction of fevers

**Figure 4:**
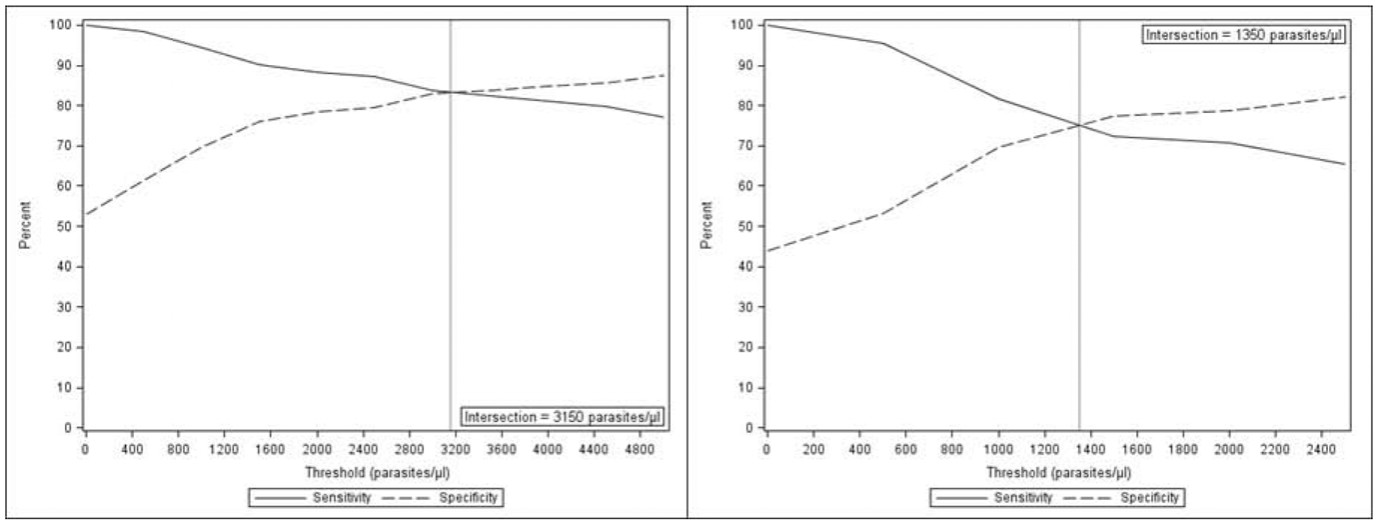
Malaria case definition using objective and subjective fever according to the season. a. Sensitivity and specificity of alternative parasite threshold for malaria case definition in the high transmission season using objective (> = 37.5 degrees C) and subjective fever. b. Sensitivity and specificity of alternative parasite threshold for malaria case definition in the low transmission season using objective (> = 37.5 degrees C) and subjective fever.

Malaria attributable fraction of fever was 41.9% in the high season and 34.5% in the low season, where fever was defined as reported fever in the past 24 hours and measured temperature ≥37.5°C. The alternative parasite thresholds for the malaria case definition that achieved optimal sensitivity and specificity (70–80%) were 3150 parasites/µl in the high and 1350 parasites/µl in the low season (Figure 4a & 4b).

**Figure 5:**
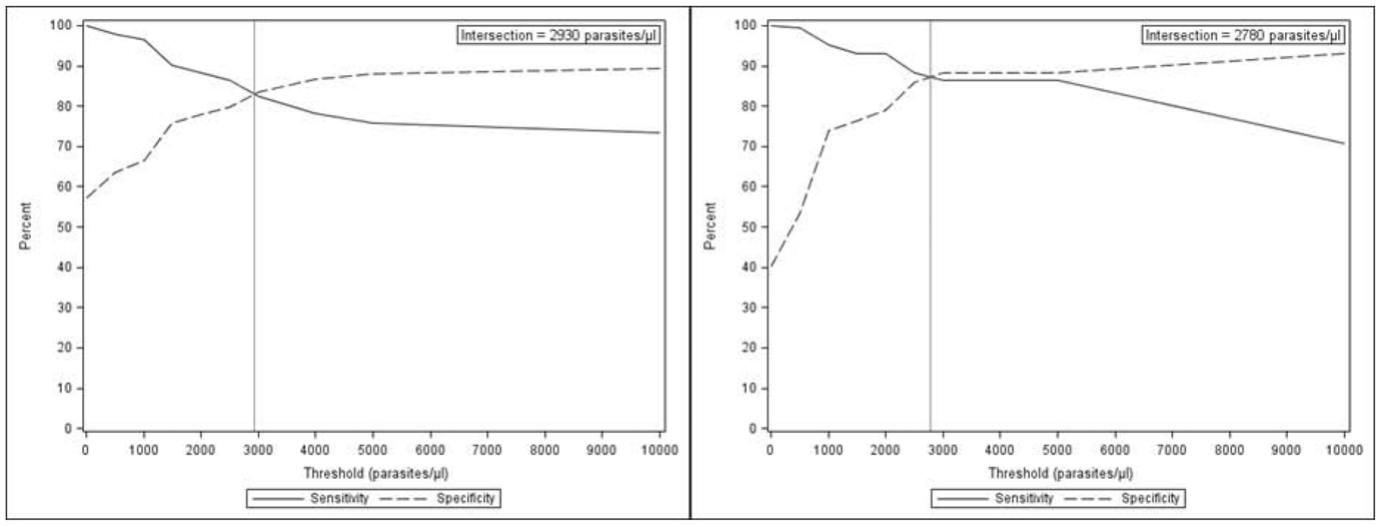
Malaria case definition using objective fever according to the season. a. Sensitivity and specificity of alternative parasite threshold for malaria case definition in the high transmission season using objective (> = 37.5 degrees C) fever. b. Sensitivity and specificity of alternative parasite threshold for malaria case definition in the low transmission season using objective (> = 37.5 degrees C) fever.

The objective definition of fever (axillary temperature ≥37.5°C) yielded a malaria attributable fraction of fever of 59.6% in the high and 17.2% in the low transmission season. The thresholds that achieved sensitivity and specificity were 2930 parasites/µl in the high and 2780 parasites/µl in the low season (Figure 5a & 5b).

## Discussion

In this cohort of children under five years living in Burkina Faso, cumulative incidence by active case detection over one year follow-up exceeded 90% in the youngest children. Sixty-five percent of malaria episodes were detected during the rainy season. These data characterize the Saponé district as a stable malaria transmission area with marked seasonality [10], [15]. Despite introduction of effective medications and vector controls to the region, malaria burden in the region remains significant and consistent with other sub-Saharan African countries [16]–[19].

### Incidence

*Plasmodium falciparum* is the most common vector of malaria in the Balonghin region. Clinical malaria incidence was highest among children under three years and showed graded decline through five years, a pattern consistent with previous reports [18], [20]. Continued exposure to malaria among infants and children living in this and other endemic regions may contribute to development of partial immunity in early life [20].

Despite high all year-round endemicity, clinical malaria was markedly more common during the rainy months of June-November, a pattern previously reported in Burkina Faso [15], [21], [22]. Age and seasonal patterns of malaria morbidity were consistent among alternative parasite thresholds for the malaria case definition.

### Active and passive surveillance

Age and seasonal trends also persisted among passive (PCD) and active case detection (ACD) cohorts; however, fever and clinical malaria incidence detected by ACD were approximately twice that by PCD.

These data suggest that as many as half of malaria episodes identified by active follow-up may be undetected by passive surveillance in this and comparable settings. This expected advantage of ACD highlights the common occurrence of unattended symptomatic malaria. Variation among cohorts may also in part reflect study design limitations. Passive surveillance outcomes may be underestimated due to alternative care seeking from non-study clinics or traditional healers or use of anti-malarials from the local market. Moreover, parents of ACD children may have been more likely to seek medical care for symptomatic illness as encouraged by field workers during regular home visits; by contrast, parents of PCD children received this instruction only at enrolment. It is also possible that time at risk for malaria, particularly among ACD children, is in part overestimated where chemoprophylaxis due to antimalarial treatment may have extended beyond the 28 day censoring window imposed after each observed episode. Refinement of censoring windows according to treatment duration information not available in this study may be expected to minimize associated inflation of time at risk, yielding higher than current incidence estimates.

### Malariometric indicators by cross sectional survey

Prevalence of clinical malaria in the ACD cohort ranged from 10–13%, and prevalence of *P.f.* infection from 49–58% among transmission seasons. Age was not consistently associated with prevalence of clinical malaria, *P.f.* infection or splenomegaly. Prevalences of *P.f.* infection, gametocyte carriage, and splenomegaly were slightly higher in the low relative to high transmission season. These data may reflect protection provided by longitudinal active surveillance, in which frequent contacts initiated following the low season survey prompted early diagnosis and treatment and potentially chemoprophylaxis effect particularly through the subsequent high season. Prevalence of malariometric indices may be considered underestimated as a result of this potentially artificially healthy ACD cohort.

### Time to the first episode

Time to first malaria episode from the low season survey was lowest among ages 0–2 years, consistent with previously described vulnerability associated with the loss of protection by maternal immunoglobulin following the first six months of life. Cumulative incidences of malaria were also highest among this age group, potentially reflecting a critical period for induction of partial immunity associated with repeat malaria exposure in early life. Children 3–5 years were more likely to escape infection during the high transmission season, suggesting development of immune protection prior to our observation.

### Fraction attributable

Estimated proportion of fever attributable to malaria parasitaemia varied substantially by malaria case definition and were nearly two fold greater in the high relative to low transmission season. These data yielded optimal parastemia thresholds for malaria case definition ranging from approximately 3000 parasites/µl in the high and 1350 parasites/µl in the low season. Marked dependence of these characterizations on season and case definition have previously been described among children living in an endemic areas of Mali and Ghana [12], [23]. Variation in estimates according to fever definition was more recently characterized among cohorts living in low to moderate transmission areas of rural Kenya Olotu [14]. Among the current ACD cohort, nearly 80% of cases with objective fever and 65% of the cases with subjective or objective fever identified during the low season were in fact not malaria. Moreover, during the high transmission season, subjective or objective fever was associated with a consistently lower malaria attributable fraction of fever than measured temperature only. These data suggest an advantage of objective fever in defining malaria outcomes due to low specificity of subjective fever. Consideration of optimal parasitemia thresholds for malaria case definitions, particularly during the high season, may also avoid overestimation of malaria burden due to low specificity of low parasitemia levels.

Practicians in this region may be advised to investigate alternative etiology of fevers given parasitemia <1350 in the dry or <3150 µl in the rainy season, the optimal thresholds derived from the favored objective fever definition.

## Conclusion

In this high and seasonal transmission region of Burkina Faso, burden of malaria was highest among the youngest children, who may be considered the most appropriate target for malaria vaccine candidates. Active case detection identified nearly twice the incidence of malaria episodes relative to the passive system, highlighting the common occurrence of potentially clinically important unattended illnesses and resulting advantage of enhanced surveillance methods in estimation of disease burden. The pyrogenic threshold for malaria case definition differed markedly by season, suggesting that alternative etiologies of fever should be considered in the presence of low parasitemia levels particularly during the low transmission season. Characterization of malaria epidemiology in the Balonghin region reinforces the urgency of improved access to currently available medication and vector control tools, while setting the stage for conduct of early phase vaccine trials in local field sites.
